# Purified β-glucans of Different Molecular Weights Enhance Growth Performance of LPS-challenged Piglets via Improved Gut Barrier Function and Microbiota

**DOI:** 10.3390/ani9090602

**Published:** 2019-08-24

**Authors:** Junqiu Luo, Daiwen Chen, Xiangbing Mao, Jun He, Bing Yu, Long Cheng, Dafu Zeng

**Affiliations:** 1 Institute of Animal Nutrition, Sichuan Agricultural University, and Key Laboratory of Animal Disease Resistance Nutrition Ministry of Education, Chengdu 611130, Sichuan, China; 2 Faculty of Veterinary and Agricultural Sciences, The University of Melbourne, Dookie Campus, VIC 3647, Australia; 3 Sichuan Synlight Biotech Ltd., Chengdu 610041, Sichuan, China

**Keywords:** β-glucan, barrier function, microbiota, piglets

## Abstract

**Simple Summary:**

Beta-glucan is currently under consideration as an alternative to in-feed antibiotics for the sustainable pig production industry in China. Modulating intestinal function by β-glucan treatment in young pigs is one potential way of decreasing disease susceptibility and presumably increasing growth performance. In the present study, as a newly developed commercial product, β-glucans have proved to modulate gut function, and have improved growth performance in lipopolysaccharide (LPS)-challenged piglets. The present study aimed to determine the mechanisms involved inβ-glucan of low and high molecular weight mediated growth alterations in weaned piglets. The results confirmed that β-glucans isolated from *Agrobacterium* sp. ZX09 could improve growth performance in weaned piglets and they showed intestinal modulatory properties via different mechanisms in regulating the mucosal barrier function and microbial populations between two different molecular weight β-glucans.

**Abstract:**

This study investigated β-glucan derived from *Agrobacterium* sp. ZX09 with high (2000 kDa) and low (300 kDa) molecular weight (MW) to compare their effects on growth performance and gut function in LPS-induced weaned piglets. Changes in jejunal morphology, mucosal barrier function, microbial populations, and fermentation in the piglets were determined. Data showed that β-glucan prevented body weight loss in LPS challenged piglets. Supplementation with both β-glucan fractions improved jejunal morphology. Compared to low MW, β-glucan of high MW generally up-regulated transcripts of *ZO-1*, *MUC1*, and *MUC2 in jejunal mucosa to a lesser extent. Mucosal D-lactate, diamine oxidase, and anti-oxidation index were effectively resumed in β-glucan treatment. Both β-glucan diets provoked the emergence of a balanced microbiota and a richer concentration of volatile fatty acids in the colon. The richest community of bifidobacterium* and concentration of butyrate emerged after feeding β-glucan with high MW. Results suggested that the effect of *Agrobacterium* sp. ZX09 β-glucans on the gut-modulatory function is largely linked to their MW. Low MW β-glucan mainly improved the mucosal barrier function in the jejunum, while high MW β-glucan had profound effects on the microbial community and fermentation in the hindgut of piglets.

## 1. Introduction

Due to underdevelopment of intestinal function at weaning, weaning pigs are susceptible to gastrointestinal pathogens [1,2]. Modulation of gut morphology and immunity following β-glucans treatment in young pigs is becoming one of the potential strategies to counter disease [3]. It has been reported that β-glucans can be utilized by microflora to form short chain fatty acids (SCFA), which has been proved to improve intestinal health and provide therapeutic strategies for pathogens infection [4,5].

Previously, most studies referred to the stimulatory properties of glucans presenting in the cell walls of algae, fungi, yeast, cereal grains, which have been performed in rats [6], chickens [7], fish [8], pigs [9], and cattle [10]. However, no substantial consistent effects of dietary glucans were found on the intestinal function. The divergence might be caused by the quality of variant glucans used in the study.

Our previous work has shown that β-glucans has multiple functional properties, such as purity, structure, and molecular weight (MW) [11]. As shown in Table 1, the purity of β-glucan in different sources varies dramatically. It has been demonstrated that a difference in molecular structure or changes in MW may influence their antioxidant effects [12]. In addition, β-glucans with different MW elicit immune-modulating responses by different mechanisms [13]. However, its intestinal-modulatory mechanism remains unknown.

Lipopolysaccharide (LPS)-induced experimental model was used in this study, and we hypothesized that β-glucans derived from *Agrobacterium* sp. ZX09 may aid in gut health during periods of intestinal imbalance induced by LPS, and that the intestinal modulatory function may depend on the varied molecular weight of β-glucans. Hence, the aim of the present study was to investigate the effect of β-glucan with different MW on growth performance and intestinal function in lipopolysaccharide (LPS)-challenged piglets.

## 2. Materials and Methods

### 2.1. β-Glucan Samples

*Agrobacterium* sp. ZX09 (Salecan^®^) was kindly provided by Synlight Bio Co. Ltd. of Sichuan, Chengdu, China. The method of β-glucan preparation was previously described [14]. The total sugar content of the fraction was determined by the phenol-sulfuric acid method, using glucose for the standard curve [15], and purity of the purified β-glucan was more than 90%. The average MW of purified β-glucan was about 2000 kDa and 300 kDa in high and low MW of β-glucan, respectively.

### 2.2. Animals and Diets

The animal protocol was approved by the animal care and use committee of Sichuan Agricultural University (Chengdu, China). The experiment was conducted at the animal experiment center of Sichuan Agricultural University (Yaan, China). A total of 32 crossbred (Duroc × Large White × Landrace) barrows weaned at 21 days of age were housed and fed individually in a temperature-controlled room, maintained at 25–28 °C. All piglets were allowed ad libitum access to feed and water. At 0800 h of days 1, 21, and 28, the body weight (BW) and feed intake of all pigs were measured.

Antibiotic-free diets were formulated to meet national research council-recommended nutrient requirements for pigs (NRC 2012). The experimental diets consisted of a control mash diet based on maize and soybean meal or the same diet plus 50 mg/kg addition of high (HG) or low (LG) molecular weight β-glucan, respectively. The ingredient composition and chemical analysis of basal diet are presented in Table 2.

### 2.3. Experimental Design

After 3 days of acclimatization, thirty-two weaned piglets were randomly assigned to one of three diets supplemented with 0 (control, n = 16) and 50 mg high (HG, n = 8) and low (LG, n = 8) MW β-glucan per kg of diet for 21 days. The LPS-induced pathogens invading model was established according to the method previously described [16]. On day 22, half of the piglets (n = 8) on the basal diet and the piglets in the HG (n = 8) and LG (n = 8) group received an intraperitoneal injection (150 μg/kg BW) of LPS from *Escherichia coli*, serotype O55:B5 (L2880 Sigma-Aldrich, St. Louis, MO, USA), and the other half of the piglets (n = 8) on control group were injected with sterile saline solution as the diluent for LPS. After 3 days post-infusion of LPS (from day 22 to day 24), fecal consistency was recorded and scores of diarrheas were calculated according to the method previously described [17].

### 2.4. Sampling and Analyses

On day 28, following weighing, blood samples were collected from each pig via jugular venipuncture. The blood sample was subsequently centrifuged for 15 min at 3000× *g* to harvest serum and then stored at −80 °C until further analysis. Piglets were then anesthetized (with C_3_H_2_ClF_5_O) and euthanized by intravenous administration (jugular vein) of 4% sodium pentobarbital solution (40 mg/kg BW). Then, the small intestine was removed, and the jejunum (proximal half of the small intestine) was quickly isolated, flushed with ice-cold saline, and then divided into two sections. One section (about 2 cm in length) was placed in 10% phosphate-buffered formalin for histologic analysis, whereas a mucosal sample from the other section (approximately 18 cm in length) was scraped by slide, frozen in liquid nitrogen, and stored at −80 °C for real-time quantitative PCR (RT-PCR) and anti-oxidant analysis. Additionally, the mid-colon tissues were removed and the digesta sample (approximately 10 g) of each pig was stored at −80 °C for microflora and volatile fatty acid analysis.

### 2.5. Jejunal Morphology 

Formalin-fixed jejunal cross-sections were embedded in paraffin wax, and 5 μm slides were cut and stained with hematoxylin and eosin. Villus height (the apex of the villus to the villus–crypt junction) and crypt depth (villus–crypt junction to the base of the crypt) were measured at 40× magnification using an image processing and analysis system (Leica Imaging Systems Ltd., Cambridge, UK). At least 10 well-oriented intact villi and crypts were examined from each pig.

### 2.6. Mucosal D-Lactate, Diamine Oxidase, and Anti-Oxidation Index 

Mucosal D-lactate was measured using a commercial kit (Beijing Leadman Biochemistry Co., Beijing, China) and a Beckman CX4 chemistry analyzer (Beckman Coulter, Brea, CA, USA). The indicators of anti-oxidant status total antioxidant capacity, T-AOC; catalase, CAT; glutathione peroxidase, GSH-Px; malondialdehyde, MDA; superoxide dismutase, SOD, diamine oxidase, DAO) were determined by using corresponding reagent kits (Jiancheng Bioengineering Institute, Nanjing, China) according to the protocol [1].

### 2.7. Mucosal mRNA Expression of ZO-1, Occludin, Claudin-1, and Mucin

Total RNA was extracted from a snap-frozen jejunal mucosa sample with Trizol reagent (Invitrogen, Carlsbad, CA, USA) and quantified by measuring absorption at 260 nm. The RNA was reverse-transcribed using a high-capacity cDNA reverse transcription kit (Applied Biosystems, Foster City, CA) with a 3 μg RNA sample according to the manufacturer’s instructions, and then amplified by PCR. Primers (Sangon Biotech, Shanghai, China) used for amplification of target *ZO-1*, *Occludin*, *Claudin-1*, *MUC1*, *MUC2*, and housekeeping *β-actin* genes are shown in Table 3. Amplification was carried out in a total of 25 μL, which contained 12.5 μL QuantiFast SYBR Green Mastermix (Applied Biosystems Inc.), forward and reverse primers (5 μmol/L), and 1 μL cDNA reaction mixture by an Option DNA engine (Bio-Rad), with the following PCR amplification conditions: 95 °C for 10 s, 40 cycles at 95 °C for 5 s, 60 °C for 25 s, followed by a final single extension step of 72 °C for 5 min. Melt curve analysis was conducted to validate the specificity of the primers. The expression ratio of the target genes relative to the housekeeping gene (β-actin) of each sample was calculated according to the 2^−ΔΔCt^ method [18]. All determinations were performed in duplicate.

### 2.8. DNA Extraction and Real-Time PCR Analysis of Colonic Bacteria

The genomic DNAs were extracted from 0.1 g of digesta samples using a commercially available rapid bacterial genomic DNA isolation kit (Sangon Bitech, Shanghai, China) for pigs. Based on the genetic sequence of 16 s rRNA of bacteria, the fluorescent quantitative specific primers and probe designed for *Bifidobacterium*, *Lactobacillus*, *Bacillus*, and *Escherichia coli* are presented in Table 4. All the primers and probe were commercially synthesized from Invitrogen (Shanghai, China). The copy numbers of total bacteria, *Bifidobacterium*, *Lactobacillus*, *Bacillus*, and *Escherichia coli* in the colonic samples were quantified by real-time PCR on Bio-Rad CFX96 real-time system (Bio-Rad, Hercules, CA, USA) with optical-grade 96-well plates. The reaction mixture (25 μL) for total bacteria was composed of 12.5 μL SYBR Premix Ex Taq (Takara, Dalian, China), 1 μL forward and 1 μL reverse primers (100 nM), 9.5 μL double distilled water (ddH_2_O) and 1 μL DNA. The program of reaction was as follows: 95 °C for 10 s, 40 cycles at 95 °C for 5 s, 60 °C for 25 s, followed by 95 °C for 10 s. The melting curve conditions were 95 °C for 30 s, 55 °C for 1 min, and 95 °C for 1 min. The reaction volume (20 μL) for detecting *Bifidobacterium*, *Lactobacillus*, *Bacillus*, and *Escherichia coli* was composed of 1 μL probe enhancer solution, 0.3 μL probe (100 nM), 1 μL forward and 1 μL reverse primers (100 nM), 8 μL Real Master Mix (Tiangen, Beijing, China), 7.7 μL ddH_2_O, and 1 μL DNA. The reaction protocol was 95 °C for 10 s, 50 cycles at 95 °C for 5 s, 50~60 °C for 25 s, followed by 95 °C for 10 s. Copies per sample were calculated with Ct-values and standard curves. The respective standard curves were generated by constructing standard plasmids containing the 16 s rRNA genes, as previously described [19,20]. Briefly, Deoxyribonucleic acid concentrations of standard plasmids were detected by a spectrophotometer. A series of 10-fold dilution of plasmids DNA were used to create the standard curves. Each standard curve was generated by a linear regression of the plotted points with the logarithm of template copy numbers as the abscissa and the Ct values as the ordinate. Results were presented as l g (copy numbers per gram of dry digesta).

### 2.9. Quantification of Volatile Fatty Acids (VFAs)

VFA concentrations were measured according to the method previously described [21]. Briefly, digesta sample was dissolved in 2 mL of distilled water, then centrifuged at 12,000× *g* for 10 min. After vortexing, the supernatants were mixed with metaphosphoric acid. The solution was centrifuged at 12,000× *g* for 10 min, then 100 uL of the upper phase were transferred to a 1 mL Eppendorf tube for GC analysis. VFA values were presented as μmoL concentration per gram of each dry digesta sample.

### 2.10. Statistical Analysis

All data from the experiment were analyzed as a complete randomized design using SAS 9.0 procedure by one-way ANOVA (SAS Institute, Cary, NC, USA). The significance between the treatment differences was identified by Duncan’s multiple comparisons test in the general linear model. Results were expressed as treatment means with their pooled SEM. A probability value of *p* < 0.05 was considered statistically significant.

## 3. Results

### 3.1. Growth Performance

During days 1–21 of the trail, compared with the control group, the average daily gain (ADG) of piglets was improved (*p* < 0.05) in LG group (Table 5). Average daily feed intake (ADFI) was higher (*p* < 0.05) in LG and HG group than in the control group. No statistically significant differences among treatments were detected (*p* > 0.05). During days 22–28 of the experiment, there were no differences in ADFI across groups (*p* > 0.05). However, LPS infusion reduced ADG of piglets (*p* < 0.05). The F:G ratio and score of diarrheas were lower with LG and HG compared with LPS-challenged piglets (*p* < 0.05).

### 3.2. Anti-Oxidation Index

Mucosal anti-oxidation index was affected across groups (Table 6). LPS injection decreased T-AOC, CAT, GSH-Px, and SOD (*p* < 0.05). Dietary β-glucan treatment, regardless of MW, prevented the LPS-reduced activity of T-AOC, CAT, and GSH-Px and normalized to the basal levels. Also, piglets fed the LG-supplemented diet had a higher SOD after LPS infusion than piglets fed the control diet (*p* < 0.05). An increase of MDA was detected following LPS challenge (*p* < 0.05); and it was attenuated by LG and HG treatment. Piglets fed LG supplemented diet had a higher value of SOD and a lower concentration of serum MDA than those fed the control diet (*p* < 0.05).

### 3.3. Morphology of Jejunal Mucosa

LPS injection modified villus height, crypt depth, and villus height:crypt depth (VH:CD), resulting in a lower villus height:crypt depth ratio in the jejunum of piglets after LPS infusion (Table 7) (*p* < 0.05). Reduced villus height and increased crypt depth were alleviated following dietary supplementation with LG and HG. In addition, LPS-challenged piglets fed with LG had greater villus height in the jejunum (*p* < 0.05), while no differences were detected in crypt depth and VH:CD in β-glucan fed group (*p* < 0.05).

### 3.4. Intestinal Barrier Function

The levels of D-lactate and DAO in serum are known as indicators of gut function. If the intestinal barrier is impaired, D-lactate and DAO concentrations in serum will increase. In the present study, increased D-lactate concentration and DAO levels in the serum of weaned piglets challenged by LPS were observed (*p* < 0.05) (Table 8). LG and HG supplemented diets reduced D-lactate and DAO concentrations of weaned piglets after LPS infusion. We also observed no difference in the concentration of D-lactate and DAO of weaned piglets between LG and HG group (*p* > 0.05).

**Figure 1:**
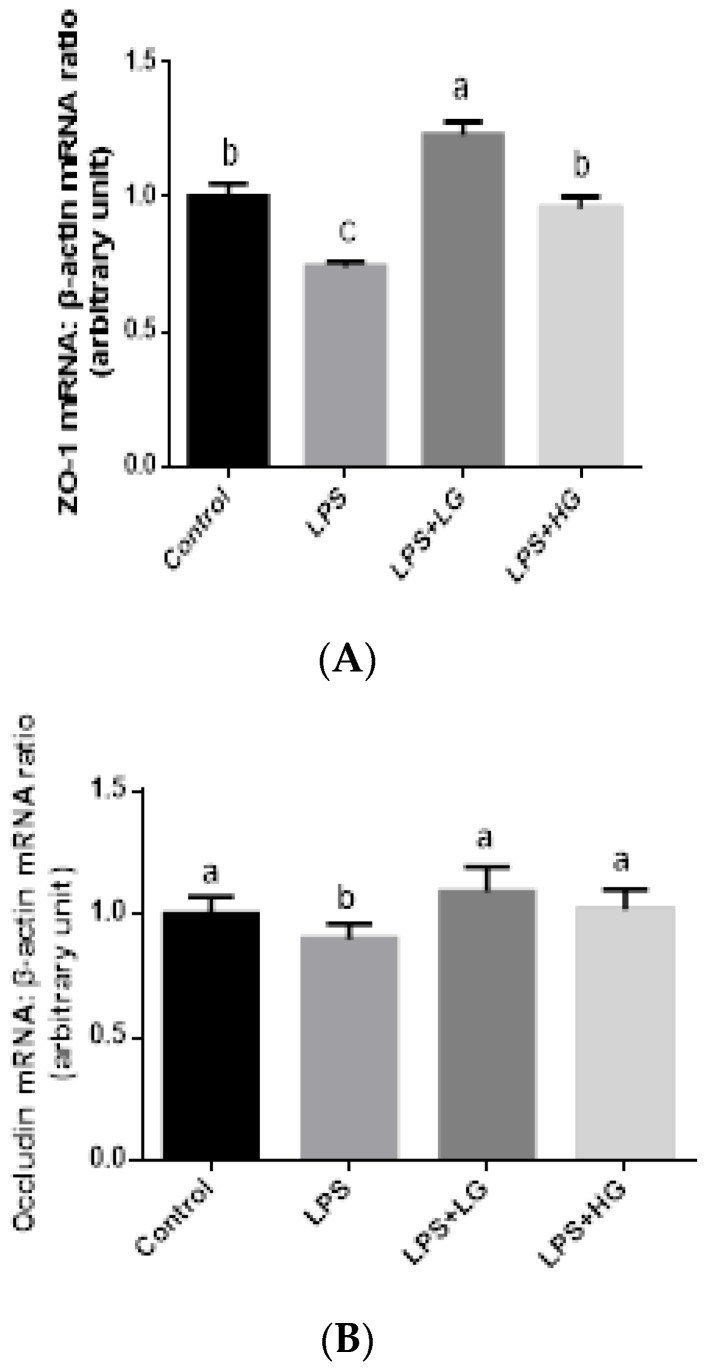

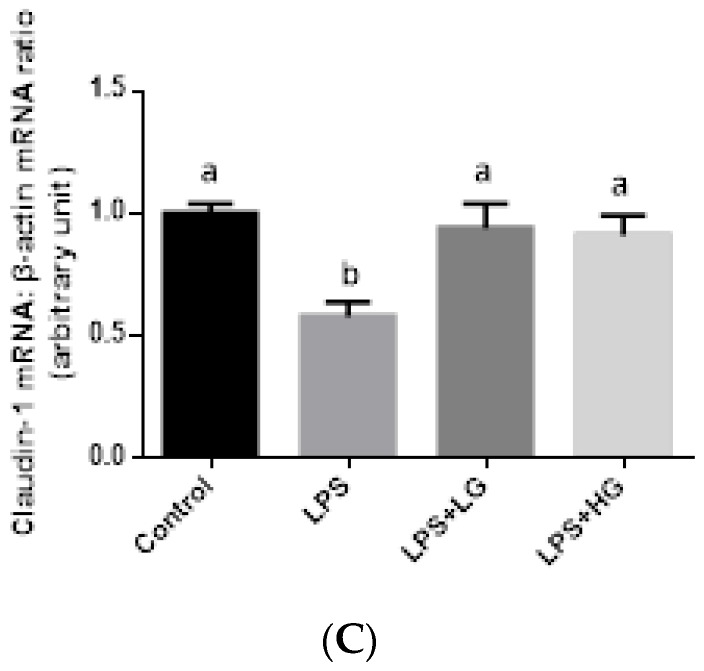
mRNA expression level of tight junction proteins in the jejunal mucosa of piglets fed different molecular weight β-glucan. Data are shown as means ± SE. (**A**) *ZO-1* mRNA expression level. (**B**) *Occludin* mRNA expression level. (**C**) *Claudin-1* mRNA expression level. Different letters indicate statistically significant differences between groups (*P* < 0.05). Control: Sterile saline solution treatment group fed basal diet; LPS+LG LPS treatment group fed basal diet; LPS + LG:LPS treatment group fed low molecular weight β-glucan; LPS + HG:LPS treatment group fed high molecular weight β-glucan.

**Figure 2:**
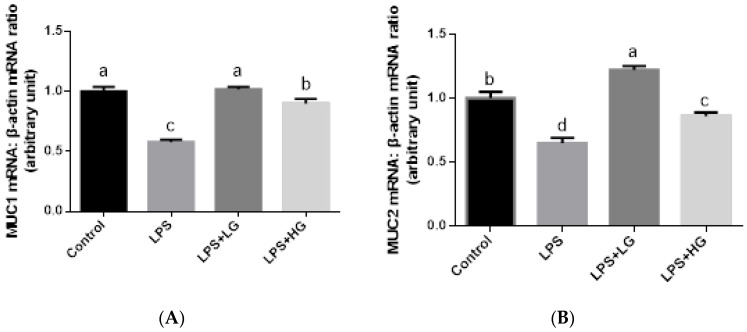
mRNA expression level of *MUC1* and *MUC2* in the jejunal mucosa of piglets fed diets with two molecular weight β-glucans. Data are shown as means ± SE. (**A**) *MUC1* mRNA expression level. (**B**) *MUC2* mRNA expression level. Different letters indicate statistically significant differences between groups (*p* < 0.05). Control: Sterile saline solution injection group fed basal diet; LPS:LPS injection group fed basal diet; LPS + LG:LPS injection group fed low molecular weight β-glucan; LPS + HG:LPS injection group fed high molecular weight β-glucan.

The impact of β-glucan of different MW on mRNA expression of tight junction proteins from jejunal mucosa is shown in Figure 1. The mRNA levels of *ZO-1*, *Occludin*, and *Claudin-1* after LPS challenge were, in general, lower than in the control group (*p* < 0.05). Additionally, β-glucan of low MW stimulated *ZO-1* expression in LPS-challenged weaned piglets (*p* < 0.05). There was no difference in *Occludin* and *Claudin-1* expressions while comparing between LG and HG supplemented group. In addition, LPS infusion declined the expression level of *MUC1* and *MUC2* (*p* < 0.05), and treatment of β-glucan could attenuate the effect of LPS challenge on Mucin gene expression in the jejunal mucosa of weaned pigs (Figure 2). Inhibited *MUC2* activation was improved by the addition LG rather than supplemented HG, when piglets were challenged by LPS (*p* < 0.05).

### 3.5. Quantitative Difference in Bacterial Groups

Quantitative PCR revealed that piglets challenged by LPS had decreased numbers of *Lactobacillus*, *Bifidobacterium*, *Bacillus*, and total bacteria than the control group (*p* < 0.05 or *p* < 0.01), but it showed increased numbers of *Escherichia coli* (*p* < 0.05) (Table 9). In addition, dietary β-glucan supplementation attenuated the impact of LPS infusion on the copy numbers of the assessed bacterial groups, particularly following HG ingestion. Compared with the control group, HG treatment increased the number of *Bifidobacterium* and *Bacillus* in colonic digesta samples of LPS-challenged piglets (*p* < 0.05), whereas dietary supplementation of LG did not show this kind of positive efficacy.

### 3.6. Changes in Volatile Fatty Acid Concentration

The concentrations of acetate, propionate, butyrate, and total VFAs in colonic digesta from piglets infused with LPS were significantly declined compared with the control group (*p* < 0.05) (Table 10). Dietary β-glucan supplementation attenuated the LPS-induced decrease of VFA concentrations in colonic digesta of piglets. Meanwhile, LPS-induced piglets fed with LG had lower levels of acetate, butyrate, and total VFAs in the colonic digesta compared to the HG group (*p* < 0.05).

## 4. Discussion

A novel high purity, water-soluble extracellular β-glucans from *Agrobacterium* sp. ZX09, were used in the present study. Based on the experimental purpose, we confirmed that β-glucan isolated from *Agrobacterium* sp. ZX09 could improve growth performance in weaned piglets and clarify the comparative intestinal function-modulatory properties between the two different molecular weight β-glucans. Our results are similar to the research by Suchecka et al., but their sources (Agrobacterium sp. ZX09 vs. oat) are different [22].

β-glucans show beneficial effects on growth performance when subjects are under normal conditions [23], however, whether this effect works on pigs challenged with LPS remains unknown. In this study, low MW β-glucan increased ADG of piglets before LPS challenging, meanwhile, under immunochallenged status, β-glucan administration improved piglet growth regardless of MW when compared to the LPS group. The results are different from previous studies, which indicated that β-glucan had no effects on non-immunochallenged piglets [24]. A potential explanation for this discrepancy involves the differences of glucan source, optimal concentration, and its purity in the diet formulation in various studies. Our earlier reports demonstrated that optimal dosage of β-glucan derived from *Agrobacterium* sp. ZX09 was 50 mg per kg of diet for weaned piglets (data not shown), while the optimal dose from other β-glucans studies were different and their sources were mainly derived from the cell wall of *Saccharomycetes cerevisiae* and purified from oat. In this study, the purity of β-glucan from *Agrobacterium* sp. ZX09 was higher (>90%) than that in *Saccharomycetes cerevisiae* and oat (60–80%) [25], therefore, it may cause different animal performance. In the present study, the injection of LPS leads to release free radicals in intestinal tissue, followed by severe inflammation in the local gut section. This mechanism may relate to negative changes in mucosal barrier function, as reflected by high levels of DAO and D-lactate in serum, low expression levels of tight junction proteins and mucins, as well as a reduction of villus height and an increase of crypt depth in the jejunum of weaned pigs. We found that the mucosal antioxidant system, intestinal barrier function, and gut morphology were attenuated in LPS-challenged weaned piglets. Under LPS challenge, dietary supplementation with low and high MW β-glucan improved the digestion and absorption of nutrients (data not shown), which may clarify the increased growth performance and reduced diarrhea in piglets. These findings are in line with previous reports indicating that polysaccharides from yeast had antioxidant properties [26]. However, dietary supplementation with yeast products decreased the transcripts of *ZO-1* and *occludin* [26]. The discrepancy may be attributed to molecular conformation contained in β-glucan per se. Thus, we take MW into our consideration. In this study, it seems that low MW β-glucan displayed the most potent antioxidant effect, as indicated by higher levels of SOD and lower levels of MDA than those in the high MW β-glucan group. Similarly, low MW glucan resulted in higher levels of *ZO-1*, *MUC1*, and *MUC2* than high MW glucan did. It was demonstrated that low MW β-glucan increased expression levels of the receptors (Dectin-1 and Toll-like receptors) in jejunum and ileum of pigs [27]. High production of tight junction proteins is attributable to Dectin-1 and TLRs expression in the GI tract [28]. This could explain why low MW β-glucan would affect mucosal barrier function more effectively than high MW. Thus, we concluded that β-glucan particles with different MW may use differential mechanisms for tight junction proteins and mucin secretion in jejunal mucosa of weaned piglets that may impact gut integrity and mucosal barrier function.

Balanced microbiota in the gut has a great influence on nutrient metabolism and intestinal health [29]. In the current study, we speculate that early weaning at 21 days of age may account for the low number of *lactobacillus* and *bifidobacterium*, as shown in Table 9. In contrast, previous research showed piglets weaned at 28 days had relatively higher numbers of these beneficial bacteria [30]. Early weaning causes major stress to gut microflora profile formation, resulting in microbiota dysfunction and malnutrition in piglets [31]. This study indicated that the LPS challenge changed the selected bacterial populations and feeding β-glucan diet to piglets positively modified the microbiota and their metabolites in the colon. Piglets fed 100 mg/kg β-glucan from *Saccharomyces cerevisae* exhibited decreased numbers of *Escherichia coli* with no effects on *Lactobacillus* and *Bifidobacterium* communities in their fecal microbiota [23]. This is consistent with the reduction in colonic enterobacteriaceae populations, when piglets were fed with 300 mg/kg laminarin derived from Laminaria digitata [32]. Importantly, this study on β-glucan with different MW revealed that high MW β-glucan had more positive effects on *Bifidobacterium* and *Bacillus* than low MW β-glucan. In this study, β-glucan is derived from *Agrobacterium* sp. ZX09, which has a molecular structure that is distinct from the *Saccharomyces cerevisae* and laminarin extracts, however, it possesses not only various biological functions, but also behaves as potential substrates for bacteria phenotype. Also, due to higher polymerization, a higher MW β-glucan would not be digestible across the prior digestive tract. When arriving at the hind gut, β-glucan exerts promotion of beneficial bacteria and suppression of deleterious bacteria. This supports the concept of improved bacterial population balance induced by *Agrobacterium* sp. ZX09 glucan of high MW in this study.

## 5. Conclusions

Improved growth performance in response to *Agrobacterium* sp. ZX09 β-glucans supplementation in weaned piglets infused with LPS may be explained by the amplified intestinal function. The effect of the β-glucans treatment on intestinal modulation is linked to their molecular weight. Low molecular weight β-glucan provoked the emergence of a more improved barrier function in the jejunum, while high molecular weight β-glucan had the most profound effect on the microbial community and fermentation in the hindgut of piglets. This insight might provide a novel nutrition strategy to maintain the intestinal health of young piglets and find an alternative to in-feed antibiotics.

## Figures and Tables

**Table 1:** Structure and content of beta-glucan preparations.

Beta-Glucan Source	MW (kDa)	Content (%)	Structure
Yeast	5–80	≤30%	β-1,3/1,6 β-1,3/1,4
Oat	5–250	≤85%	β-1,3/1,4
Algal	<5	≤80%	β-1,3/1,6
*Agrobacterium* sp. ZX09	200–3000	≥90%	β-1,3

**Table 2:** The composition and nutrient content of basal diet (as-fed basis).

Ingredients	Composition, g/kg
Corn	301.8
Extruded corn	290.0
Fish meal	40.0
Whey powder	40.0
Soybean meal	107.6
Extruded full-fat soybean	100.0
Soy protein concentrate	50.0
Wheat bran	20.0
Corn starch	5.0
L-Lysine·HCL (78%)	3.3
L-Threonine (98.5%)	1.5
DL-Methionine (99%)	0.9
L-Tryptophan	0.3
Choline chloride	1.0
Sodium chloride	3.0
Calcium carbonate	7.0
Dicalcium phosphate	5.5
Soybean oil	17.8
Vitamin premix ^1^	0.3
Mineral premix ^2^	5.0
Nutrient composition, g/kg	
Digestible energy ^3^, MJ/kg	14.83
Crude protein ^4^	205.6 ± 3.51
Total lysine ^4^	13.5 ± 0.20
Total methionine and cysteine ^3^	7.4
Total tryptophan ^3^	2.2
Total threonine ^3^	7.9
Calcium ^3^	8.0
Phosphorus available ^3^	4.0

^1^ Provided the following per kg of diet: Vitamin A, 8000 IU; Vitamin D_3_, 1500 IU; Vitamin E, 25 IU; Vitamin K_3_, 2.0 mg; Vitamin B_1_, 2.0 mg; Vitamin B_2_, 5.0 mg; Vitamin B_6,_ 4.0 mg; Vitamin B_12_, 0.1 mg; Nicotonic, 25 mg; Pantothenic, 12 mg; Folic acid, 0.75 mg; Biotin, 0.2 mg. ^2^ Provided the following per kg of diet: Fe (FeSO_4_·7H_2_O),100 mg; Cu (CuSO_4_·5H_2_O), 6 mg; Zn (ZnSO_4_·7H_2_O), 100 mg; Mn (MnSO_4_·H_2_O), 4 mg; Se (Na_2_SeO_3_·5H_2_O), 0.35 mg; I (KI), 0.14 mg. ^3^ Calculated values. ^4^ Measured values.

**Table 3:** Primers used for quantitative RT-PCR of mucosal barrier function.

Gene	Forward Primer (5′ to 3′)	Reverse Primer (5′ to 3′)	Accession Number.	Product Length
*ZO-1*	CAGCCCCCGTACATGGAGA	GCGCAGACGGTGTTCATAGTT	XM_005659811	114bp
*Occludin*	CTACTCGCTCAACGGGAAAG	ACGCCTCCAAGTTACCZCTG	NM_001163647.2	158bp
*Claudin-1*	TCTTAGTTGCCACAGCATGG	CCAGTGAAGAGAGCCTGACC	NM001244539	106bp
*Mucin 1*	GTGCCGCTGCCCACAACCTG	AGCCGGGTACCCCAGACCCA	XM_001926883.4	141bp
*Mucin 2*	GGTCATGCTGGAGCTGGACAGT	TGCCTCCTCGGGGTCGTCAC	XM_003122394.1	181bp
*β-actin*	TCTGGCACCACACCTTCT	TGATCTGGGTCATCTTCTCAC	DQ178122	114bp

**Table 4:** Primers/probes for real-time PCR of bacteria.

Item	Primers/Probes and Sequence (5′–3′)	Product Length
Total bacteria	Eub338F: ACTCCTACGGGAGGCAGCAG	200bp
	Eub518R: ATTACCGCGGCTGCTGG	
*Lactobacillus*	F: GAGGCAGCAGTAGGGAATCTTC	126bp
	R: CAACAGTTACTCTGACACCCGTTCTTC	
	P: (FMA)AAGAAGGGTTTCGGCTCGTAAAACTCTGTT(BHQ-1)	
*Bifidobacterium*	F: CGCGTCCGGTGTGAAAG	121bp
	R: CTTCCCGATATCTACACATTCCA	
	P: (FMA) ATTCCACCGTTACACCGGGAA(BHQ-1)	
*Bacillus*	F: GCAACGAGCGCAACCCTTGA	92bp
	R: TCATCCCCACCTTCCTCCGGT	
	P: (FMA)CGGTTTGTCACCGGCAGTCACCT(BHQ-1)	
*Escherichia coli*	F: CATGCCGCGTGTATGAAGAA	96bp
	R: CGGGTAACGTCAATGAGCAAA	
	P: (FMA)AGGTATTAACTTTACTCCCTTCCTC(BHQ-1)	

**Table 5:** Growth performance of piglets fed different molecular weight β-glucan.

Item	Control	LPS ^1^	LG ^2^	HG ^3^	SEM	*p*-Value
1–21d ^6^						
ADG(g)	305 ^a^		371 ^b^	348 ^ab^	29.12	0.048
ADFI(g)	538 ^a^		597 ^b^	582 ^b^	24.73	0.039
F/G	1.78		1.63	1.67	0.12	0.338
			LPS + LG ^4^	LPS + HG ^5^	SEM	*p*-value
22–28d ^7^						
ADG(g)	415 ^a^	325 ^b^	390 ^a^	411 ^a^	27.47	0.035
ADFI(g)	656	602	621	637	29.35	0.55
F/G	1.60 ^a^	1.88 ^b^	1.58 ^a^	1.56 ^a^	0.09	0.027
Score of diarrheas	3.50 ^a^	7.50 ^b^	4.75 ^a^	5.00 ^a^	0.55	0.041

^1^ LPS, lipopolysaccharide. ^2^ LG, low molecular weight glucan. ^3^ HG, high molecular weight glucan. treatmen^4^ LPS + LG, LPS treatment group fed low molecular weight β-glucan. ^5^ LPS + HG, LPS treatment group fed high molecular weight β-glucan. ^6^ 1–21d: n = 16 replicates in control group; n = 8 replicates in LG group; n = 8 replicates in HG group.^7^2–28d: ^a,b^ Mean values within a row with unlike superscript letters were significantly different (*p* < 0.05).

**Table 6:** Mucosal anti-oxidation index of piglets fed different molecular weight β-glucan.

Item	Control	LPS ^1^	LPS + LG ^2^	LPS + HG ^3^	SEM	*p*-Value
T-AOC (U/mg prot)	3.26 ^a^	1.84 ^b^	3.86 ^a^	3.72 ^a^	0.23	<0.01
CAT (U/mg prot)	3.58 ^a^	2.69 ^b^	3.88 ^a^	3.43 ^a^	0.32	0.039
GSH-Px (U/mg prot)	50.86 ^a^	40.69 ^b^	53.21 ^a^	51.64 ^a^	2.05	<0.01
SOD (U/mg prot)	51.18 ^b^	29.35 ^c^	67.14 ^a^	54.22 ^b^	3.95	0.025
MDA (nmol/mg prot)	2.33 ^b^	3.57 ^a^	1.59 ^c^	2.41 ^b^	0.21	0.047

^1^ LPS, LPS treatment group fed control diet. ^2^ LPS + LG, LPS treatment group fed low molecular weight β-glucan. ^3^ LPS + HG, LPS treatment group fed high molecular weight β-glucan. ^a,b,c^ Mean values within a row with unlike superscript letters were significantly different (*p* < 0.05). n = 8 replicates per group.

**Table 7:** Jejunal morphology of piglets fed different molecular weight β-glucan.

Item	Control	LPS ^1^	LPS + LG ^2^	LPS + HG ^3^	SEM	*p*-Value
Villus height (μm)	319 ^b^	269 ^c^	368 ^a^	325 ^b^	6.11	0.031
Crypt depth (μm)	178 ^b^	213 ^a^	177 ^b^	178 ^b^	3.32	0.042
VH:CD ^4^	1.80 ^a^	1.26 ^b^	2.09 ^a^	1.84 ^a^	0.05	0.040

^1^ LPS, LPS treatment group fed control diet. ^2^ LPS + LG, LPS treatment group fed low molecular weight β-glucan. ^3^ LPS + HG, LPS treatment group fed high molecular weight β-glucan. ^4^ VH:CD, Ratio of villus height to crypt depth. ^a,b,c^ Mean values within a row with unlike superscript letters were significantly different (*p* < 0.05). n = 8 replicates per group.

**Table 8:** Serum D-lactate and diamine oxidase (DAO) of piglets fed different molecular weight β-glucan.

Item.	Control	LPS ^1^	LPS + LG ^2^	LPS + HG ^3^	SEM	*p*-Value
D-lactate (μg/mL)	8.87 ^b^	10.99 ^a^	8.46 ^b^	8.75 ^b^	0.45	<0.01
DAO (U/L)	14.68 ^b^	17.14 ^a^	14.15 ^b^	13.07 ^b^	0.40	0.026

^1^ LPS, LPS treatment group fed control diet. ^2^ LPS + LG, LPS treatment group fed with low molecular weight β-glucan. ^3^ LPS + HG, LPS treatment group fed with high molecular weight β-glucan. ^a,b^ Mean values within a row with unlike superscript letters were significantly different (*p* < 0.05). n = 8 replicates per group.

**Table 9:** Colonic microflora of piglets fed different molecular weight β-glucan (Unit: Lg copies/gram of dry digesta).

Item	Control	LPS ^1^	LPS + LG ^2^	LPS + HG ^3^	SEM	*p*-Value
*Lactobacillus*	8.27 ^a^	7.73 ^c^	8.05 ^b^	8.25 ^a^	0.12	0.012
*Bifidobacterium*	5.66 ^b^	5.02 ^d^	5.33 ^c^	6.03 ^a^	0.28	<0.01
*Bacillus*	8.79 ^b^	8.45 ^c^	8.70 ^b^	9.47 ^a^	0.08	<0.01
*Escherichia coli*	7.47 ^b^	8.08 ^a^	7.99 ^a^	7.42 ^b^	0.15	0.011
Total bacteria	11.16 ^a^	9.61 ^c^	10.75 ^b^	11.18 ^a^	0.20	<0.01

^1^ LPS, LPS treatment group fed control diet. ^2^ LPS + LG, LPS treatment group fed low molecular weight β-glucan. ^3^ LPS + HG, LPS treatment group fed high molecular weight β-glucan. ^a,b,c^ Mean values within a row with unlike superscript letters were significantly different (*p* < 0.05). n = 8 replicates per group.

**Table 10:** Colonic volatile fatty acids (VFAs) concentrations of piglets fed different molecular weight β-glucan. (Unit: μmol/g of dry digesta).

Item	Control	LPS ^1^	LPS + LG ^2^	LPS + HG ^3^	SEM	*p*-Value
Acetate	58.02 ^a^	52.49 ^c^	55.69 ^b^	59.08 ^a^	2.90	0.021
Propionate	25.95 ^a^	22.55 ^b^	23.61 ^ab^	25.92 ^a^	1.37	0.048
Butyrate	13.21 ^b^	11.10 ^c^	13.29 ^b^	14.72 ^a^	1.13	0.033
Total VFAs	97.18 ^a^	86.14 ^c^	92.59 ^b^	99.72 ^a^	4.48	0.042

^1^ LPS, LPS treatment group fed control diet. ^2^ LPS + LG, LPS treatment group fed low molecular weight β-glucan. ^3^ LPS + HG, LPS treatment group fed high molecular weight β-glucan. ^a,b,c^ Mean values within a row with unlike superscript letters were significantly different (*p* < 0.05). n = 8 replicates per group.
